# Stem cell factor 248 shapes ILC2 transcriptional programs and promotes mucosal inflammation in allergic asthma

**DOI:** 10.3389/fimmu.2026.1843080

**Published:** 2026-07-02

**Authors:** Nobuhiro Asai, Grace K. Lombardo, Ramon Ocadiz-Ruiz, Yao Gu, Andrew J. Rasky, Dana S. Garcia, Angela J. Montoya, Sarita Montaño, Kazuma Yagi, Wendy Fonseca

**Affiliations:** Department of Pathology. University of Michigan, Ann Arbor, MI, United States

**Keywords:** allergy, asthma, c-kit, ILC2, ILCP, SCF

## Abstract

Stem cell factor (SCF), also known as Kit ligand, is a pleiotropic cytokine that signals through the c-Kit receptor to regulate cellular development, survival, and proliferation. Although SCF is classically recognized for its essential role in hematopoiesis and mast cell biology, c-Kit is also expressed by innate lymphoid cell progenitors (ILCp) and subsets of mature innate lymphoid cells (ILCs), suggesting broader immunoregulatory functions.

Group 2 innate lymphoid cells (ILC2s) are critical mediators of type 2 airway inflammation and serve as an important source of type 2 cytokines during allergic responses. We previously demonstrated increased expression of the pro-inflammatory SCF248 isoform in the lungs of mice with chronic allergic inflammation, while elevated soluble SCF levels have also been reported in patients with asthma. In the present study, we further observed that SCF248 is upregulated in the bone marrow during allergic inflammation, suggesting that SCF248 may contribute to both local and systemic regulation of allergic immune responses.

To define the role of SCF/c-Kit signaling in ILC2 biology, we first performed transcriptional profiling of SCF-deficient ILC2s, which revealed reduced expression of genes associated with cytokine signaling, activation, and effector function, including *Il4*, *Stat5b*, and *PI3K-AKT* pathway components, consistent with impaired inflammatory responsiveness. Mechanistically, pro-inflammatory and type 2 cytokines induced SCF248 expression in mesenchymal cells *in vitro*. To define its functional impact, ILC progenitors were cultured on OP9-DL1 stromal cells with upregulated SCF248 expression, which increased expression of ILC2-associated markers and the maturation program, supporting a role for SCF248 in enhancing ILC2 maturation and activation.

*In vivo* validation using tamoxifen-inducible whole-body SCF-deficient mice (SCF^fl/fl^***;*** UBC-CreERT2) in an *Alternaria alternata* model of allergic airway inflammation demonstrated that SCF deficiency reduced SCF248 expression, attenuated type 2 cytokine production, diminished lung inflammation, and decreased circulating and pulmonary ILC2 populations. Similarly, SCF248 blockade reduced allergic inflammation and altered bone marrow ILC compartments. Together, these findings identify SCF248 as a regulator of ILC2 maturation and activation, amplifying mucosal type 2 inflammation during allergic airway disease.

## Introduction

Allergic asthma is a chronic respiratory condition marked by airway inflammation, leading to shortness of breath, chest tightness, and coughing. Globally, it affects millions of people of all ages and significantly impacts the quality of life (1, 2). Allergic asthma is characterized by inflammatory responses primarily driven by the production and release of type 2 cytokines, including interleukin (IL)-4, IL-5, and IL-13, which promote airway remodeling, mucus hyperproduction, airway obstruction, and hyperresponsiveness (1, 2).

Group 2 innate lymphoid cells are now recognized as central drivers of type 2 airway inflammation in many forms of asthma, especially eosinophilic and severe disease. They respond rapidly to epithelial “alarmin” signals and produce large amounts of type 2 cytokines, thereby linking environmental triggers to chronic inflammation and exacerbations (3–6). ILC2s are markedly increased in the blood, sputum, and bronchoalveolar lavage (BAL) of patients with asthma compared with healthy controls, with higher numbers in severe and eosinophilic asthma, and are associated with worse lung function and airway eosinophilia (4, 7, 8).

ILC2s arise from fetal liver- and bone marrow-derived hematopoietic progenitors through sequential differentiation stages, including common lymphoid progenitors and multipotent PLZF^high^ ILC progenitors, which can generate ILC1, ILC2, and ILC3 subsets. Fetal-derived progenitors establish tissue ILC2 populations early in life, while bone marrow-derived progenitors contribute to their maintenance in adulthood. (9, 10).

We have investigated the role of SCF in allergic airway responses and demonstrated that it significantly affects the severity of airway responsiveness, remodeling, and chronicity (11–16). SCF is a primary cytokine involved in hematopoietic cell development of multiple lineages, as well as mast cell differentiation and activation (17, 18). SCF binds to the surface receptor c-Kit (17, 19). Endogenous SCF exists in two different isoforms, SCF220 and SCF248, both of which are transmembrane proteins. However, only the SCF248 isoform has an enzyme-cleavable domain, which allows a part of SCF248 to be released from the cell surface (soluble SCF165). Studies using Sl/Sld mice, which lack membrane-bound SCF isoforms, revealed that SCF is essential for normal growth, erythropoiesis, and immune regulation. These mice exhibit growth retardation, anemia, and reduced allergic inflammation. Reconstitution studies demonstrated distinct functions for SCF splice variants: SCF220 restored normal growth and corrected anemia, whereas SCF248 did not rescue these homeostatic defects but instead promoted inflammatory myeloid cell development. Together, these findings highlight the specialized and differential roles of SCF splice variants in hematopoietic homeostasis and inflammation (20–22).

SCF has been identified in bone marrow stromal cells, epithelial cells, endothelial cells, and fibroblasts (23–29). However, the function of SCF in ILCs within bone marrow and mucosal tissue during pulmonary allergic responses has not been thoroughly investigated. While it is not clear what role SCF/c-Kit activation plays in ILC2 cells, it may be an important pathway that promotes their differentiation, expansion, and/or survival.

The current study characterized the direct role of SCF in hematopoietic ILC2 during airway allergic inflammation using an inducible whole-body SCF-deficient mouse. Additionally, the role of the SCF248 isoform in ILCp-ILC2 differentiation was investigated. These studies further identify the important role of SCF, particularly the SCF248 isoform, in ILCp-ILC2 maturation and activation during allergic airway inflammation, especially in the bone marrow, highlighting SCF248 as a possible therapeutic target to mitigate ILC2-dependent pulmonary type 2 inflammatory responses.

## Results

### SCF deficiency alters the transcriptional landscape of bone marrow ILC2 during lung allergic inflammation

**Figure 1:**
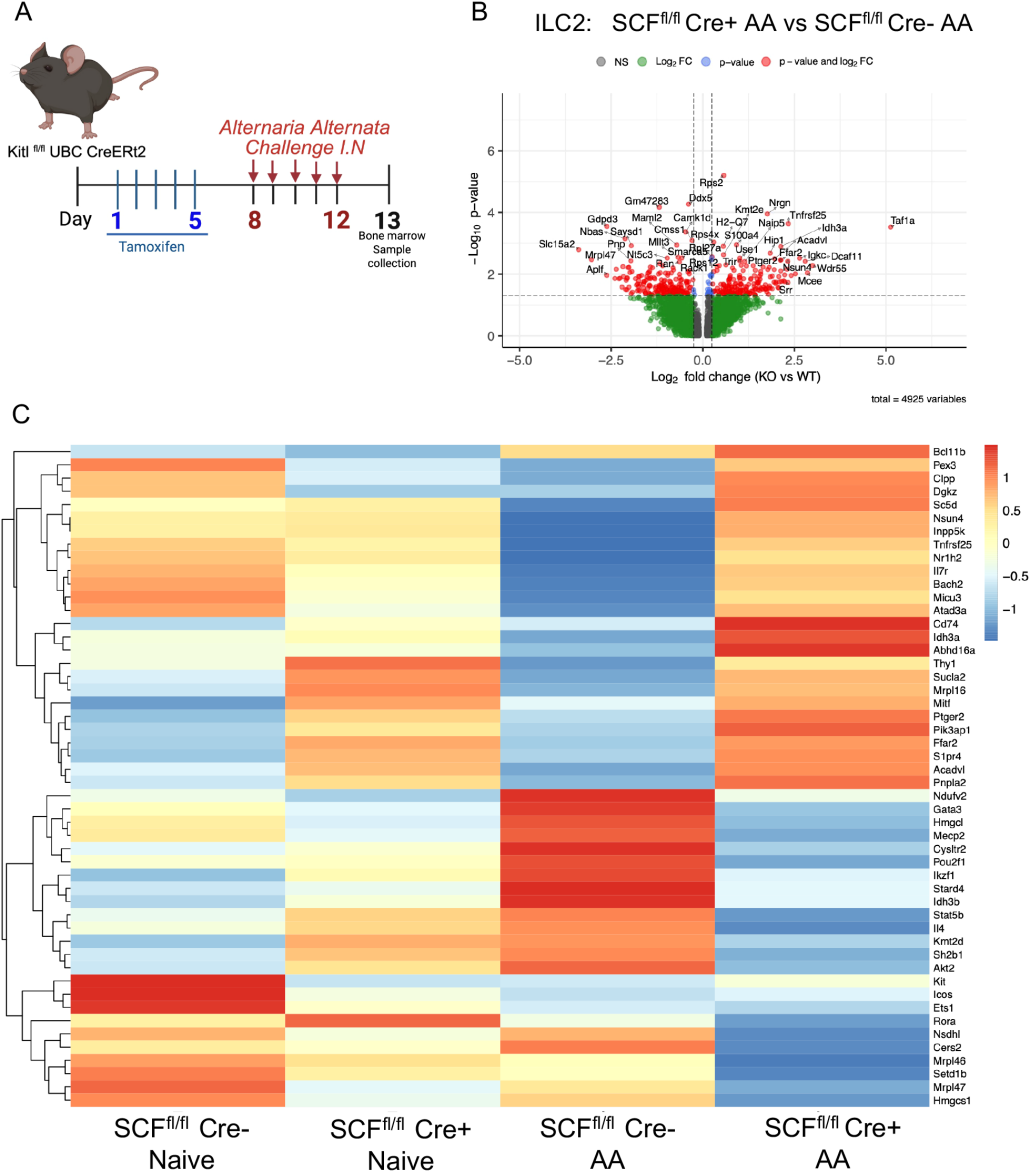
SCF deficiency alters the transcriptional landscape of bone marrow ILC2 During Lung Allergic Inflammation. **(A)** Animal model inducible SCF KO during the lung allergic model. **(B)** Volcano plot showing the different gene expressions in the ILC2 cluster SCF^fl/fl^ Cre^+^ Allergic vs. SCF^fl/fl^ Cre^-^ Allergic control. **(C)** Heat map showing Differentially Expressed Genes in Bone Marrow ILC2. N = 3 animals per group; samples from individual animals were pooled within each group for downstream analysis.

We generated whole-body tamoxifen-inducible SCF knockout (SCF^fl/fl^) mice to evaluate the role of SCF in hematopoietic ILC2 during allergic lung inflammation. To identify early transcriptional changes in the bone marrow ILC2 population, we used an acute allergic inflammation model. SCF^fl/fl^ Cre^+^ and Cre^-^ mice were treated with tamoxifen intraperitoneally and challenged with *Alternaria alternata* intranasally daily for 5 days (**Figure 1A**). To evaluate the role of SCF in hematopoietic ILC2, we performed whole-bone-marrow, unbiased scRNA-seq of SCF^fl/fl^ Cre^+^ and Cre^-^ mice, including control naive and allergic mice. Cells were clustered based on gene expression profiles, and cell types were annotated using established markers and the published literature *(*30*).* The ILC2 cluster was defined by its gene signature (*Gata3, Il7r, Il1rl1, Il17rb, Klrg1*). Differential gene expression analysis in the ILC2 cluster revealed notable transcriptional alterations in SCF^fl/fl^ Cre^+^ vs SCF^fl/fl^ Cre^-^ naive mice at baseline. Naïve SCF-deficient ILC2 exhibited reduced expression of canonical AP-1 transcription factors (*Fos, Fosb, Jun, Junb*) and activation-associated signaling regulators (*Dusp1, Rgs2, Crem*), indicating impaired basal activation priming. Furthermore, SCF deficiency reduced expression of maturation-associated genes, including *Icos, Arg1, Pik3cg, Jak1*, and *Parp14*, suggesting impaired acquisition of a fully competent effector ILC2 phenotype (**Table 1**). We next analyzed differential gene expression within the ILC2 cluster of allergic mice by comparing SCF^fl/fl^ Cre^+^ allergic vs SCF^fl/fl^ Cre^-^ groups to assess transcriptional changes during allergic inflammation. Numerous genes were significantly differentially expressed between groups, as illustrated in the volcano plot (**Figure 1B**). During allergic inflammation, ILC2 from SCF-deficient mice showed significant downregulation of critical effector and signaling-associated genes, including *Il4*, *Stat5b*, *Cysltr2*, *Sh2b1*, *Akt2*, and *Prkcd*. In contrast, the mitochondrial fatty acid oxidation gene *Acadvl* was upregulated (**Table 2**). To further characterize transcriptional changes across conditions, we generated a heatmap of selected ILC2-associated genes across naïve and allergic SCF^fl/fl^ Cre^−^ and SCF^fl/fl^ Cre^+^ mice. Unsupervised clustering revealed clear segregation between naïve and allergic samples, indicating that allergic inflammation predominantly drives ILC2 transcriptional profiles. Within the allergic condition, SCF-deficient ILC2 displayed reduced expression of key effector and signaling genes, including *Il4, Stat5b, Sh2b1, Akt2*, and *Cysltr2*, along with decreased expression of canonical ILC2 markers such as *Gata3, Icos*, and *Rora*, indicating transcriptional reprogramming in the SCFKO allergic mice (**Figure 1C**). These data indicate that SCF deficiency is associated with reduced expression of cytokines, receptors, and intracellular signaling components in ILC2, alongside increased expression of a metabolic gene involved in β-oxidation.

**Table 1:** Differentially expressed genes in the ILC2 cluster of SCF-KO (SCF^fl/fl^ Cre^+^ vs SCF^fl/fl^ Cre^-^) naive mice.

Genes	Regulation in SCF-KO	Function	Specific function in ILC2 context
Fosb	↓ Down	AP-1 family transcription factor (alternative FOS isoform). Immediate Early/Activation Genes. Can regulate inflammatory gene expression amplitude.	Often associated with prolonged stimulation responses. Fosb has been identify as a gene involving preparedness program in ILC2 during chronic allergic model (31)
Fos	↓ Down	Component of the AP-1 transcription factor complex (FOS/JUN heterodimer). AP-1 dimers (Jun–Fos/Fra) control genes involved in proliferation, survival, differentiation and inflammatory responses, and are activated by cytokines, stress and pathogens. (32)	Immediate Early/Activation Genes This gene is induced rapidly after cytokine stimulation to drive transcription of effector cytokines (IL-5, IL-13). Supports proliferation and survival and regulates metabolic reprogramming during activation. Fos has not been mechanistically analyzed in ILC2s. The downregulation of this gene may translate in reduced capacity to initiate effector transcription.
Jun	↓ Down	Jun proteins (c-Jun, JunB, JunD) are core components of the AP-1 transcription factor complex and key regulators of many immune cells. These roles show that Jun/AP-1 can drive both proinflammatory and regulatory cytokines depending on context	Jun (c-Jun/JunB/JunD) themselves are not mechanistically analyzed in ILC2s
Junb	↓ Down	Jun proteins (c-Jun, JunB, JunD) are core components of the AP-1 transcription factor complex. JunB JunB is required for clonal expansion of Th1, Th2 and Th17 cells. JunB is an essential regulator of Th17 cell identity (33, 34)	
Crem	↓ Down	cAMP Response Element Modulator. Transcription factor responding to cAMP signaling. Is a transcription factor that powerfully shapes cytokine production, especially IL-2 and IL-17A, mainly studied in T cells and NK cells (35)	For ILC2s, current literature does not report CREM expression in ILC2.
Icos	↓ Down	Inducible T-cell costimulatory (ICOS) is a crucial costimulatory molecule expressed on activated innate lymphoid cells (ILCs), particularly ILC2s and ILC3s, where it regulates homeostasis, survival, and cytokine production.	Classic costimulatory receptor on mature ILC2, required for optimal IL-5/IL-13 production, Marker of activated/mature ILC2, Supports survival and proliferation, Reduced maturation or activation competence, ICOS regulates the pool of group 2 innate lymphoid cells under homeostatic and inflammatory conditions in mice COS in innate immunity and indicate that not only cytokines, but also costimulatory pathways such as those involving ICOS, can contribute to regulate the ILC2 pool. (36)
Jak1	↓ Down	Janus Kinase 1 (Jak1). Tyrosine kinase essential for IL-4, IL-13, IL-7, IL-2, IL-9, IL-33 downstream signaling.	JAK1-dependent pathways are crucial for ILC2-driven allergic inflammationJAK1-dependent cytokines (such as IL-4 and IL-13) are key drivers of ILC2 activation, leading to allergic inflammation. (37)
Pik3cg	↓ Down	PIK3CG (encoding the PI3Kγ subunit)	PI3K/AKT signaling pathways play a critical role in regulating ILC2s. Leptin-mediated activation of this pathway enhances ILC2 cytokine production, such as IL-13, contributing to allergic inflammation. (38)
Arg1	↓ Down	Arg1 is a urea cycle enzyme that hydrolyzes L-arginine into L-ornithine and urea	Arginase 1 is an innate lymphoid-cell-intrinsic metabolic checkpoint controlling type 2 inflammation. (39) Deleting ILC-intrinsic Arg1 markedly reduces ILC2 proliferation, IL-5/IL-13 production, and type 2 lung inflammation. (40)
Orai1	↓ Down	Orai1 is an essential Ca2+ channel for cellular differentiation, facilitating extracellular Ca2+ influx known as store-operated Ca2+ entry (SOCE)	Crucial calcium release-activated calcium (CRAC) channel components that regulate ILC2 function, acting as key mediators of type 2 inflammation. Inhibiting or deleting Orai1/Orai2 reduces ILC2 cytokine production (IL-5 and IL-13) limiting their metabolic activation, and alleviating airway hyperreactivity in asthma models. (41)
IL2rb	↑ Up	β chain of IL-2/IL-15 receptor	Expressed in some activated ILC subsets, can reflect altered survival programming. (42)

**Table 2:** Differentially expressed immune-associated genes in the ILC2 cluster of SCF-KO (SCF^fl/fl^ Cre^+^ vs SCF^fl/fl^ Cre^-^) allergic mice.

Genes	Regulation in SCF-KO	Function	Specific function in ILC2 context
Ii4	↓ Down	Core type 2 effector cytokine.	Downregulated IL4 in ILC2 could be linked to reduced cytokine responsiveness and reduced effector capacity. IL-4 is required for optimal TH2 differentiation in vivo and maturation and functional identity of ILC2 (43, 44).
Stat5b	↓ Down	Stat5 (Stat5a/Stat5b) is a well-documented downstream effector of SCF/c-Kit in mast cells. (45–47)	Central to IL-7 and IL-2 signaling, survival, proliferation and effector cytokine production (48). Stat5 activating cytokines upregulate GATA3 (49).
Cysltr2	↓ Down	Cysteinyl leukotriene receptor; amplifies type 2 responses Cysteinyl leukotriene E4 (LTE4) and PGD2 are released by mast cells (50).	Cysteinyl leukotriene directly activated ILC2, reducing apoptosis and promoting type 2 cytokines, it can synergize with prostaglandin D2 and epithelial cytokines to enhance ILC2 activation and proinflammatory allergic responses (50, 51).
Sh2b1	↓ Down	Sh2b1 is an SH2 adaptor for JAK2 and several receptor tyrosine kinases, that enhance downstream signaling (52).	SCF induces association of JAK2 with c-Kit and phosphorylation of JAK2, which in turn activates STATs. Current SCF/c-Kit reviews and signaling maps highlight SH2-containing adaptors (Grb2, Gab2, Shc, PI3K, PLCγ, JAK2) but do not explicitly list SH2B1 in the SCF/c-Kit complex.
Akt2	↓ Down	PI3K-AKT metabolic survival pathway. Central to IL-7 and IL-2 signaling, survival and effector cytokine. production. AKT2 Promotes glycolysis (53).	AKT signaling is clearly a positive regulator of IL-33-driven ILC2 proliferation and type-2 cytokine. production via the PI3K-AKT axis (54). However, current ILC2 literature does not yet define AKT2-specific functions.
Prkcd	↓ Down	PKCδ is a ubiquitously expressed novel PKC isoform with context-dependent pro- and anti-inflammatory function (55).	Published work on ILC2 in Protein kinase C focuses on PKCθ (PRKCQ), which is stablish as a positive regulator of ILC2 activation during allergic lung inflammation (56).ILC2-specific role for PKCδ (PRKCD) has not yet been demonstrated.
Acadvl	↑ Up	ACADVL encodes very long-chain acyl-CoA dehydrogenase (VLCAD) a key mitochondrial enzyme for β-oxidation of very long-chain fatty acids. It supports mitochondrial protein synthesis and oxidative metabolism.	ILC2s rely on mitochondrial metabolism and fatty acid pathways, and their activation can be shaped by lipid availability and oxidative phosphorylation (57). The ACADVL-dependent processes (FAO capacity, ROS balance, mitochondrial dynamics) are all known to influence immune cell activation generally but have not been investigated the role of ACADVL in ILC2s.

### Allergic airway inflammation is attenuated in whole body SCF knockout (SCF^fl/fl;UBC-cre-ERT^) mice

**Figure 2:**
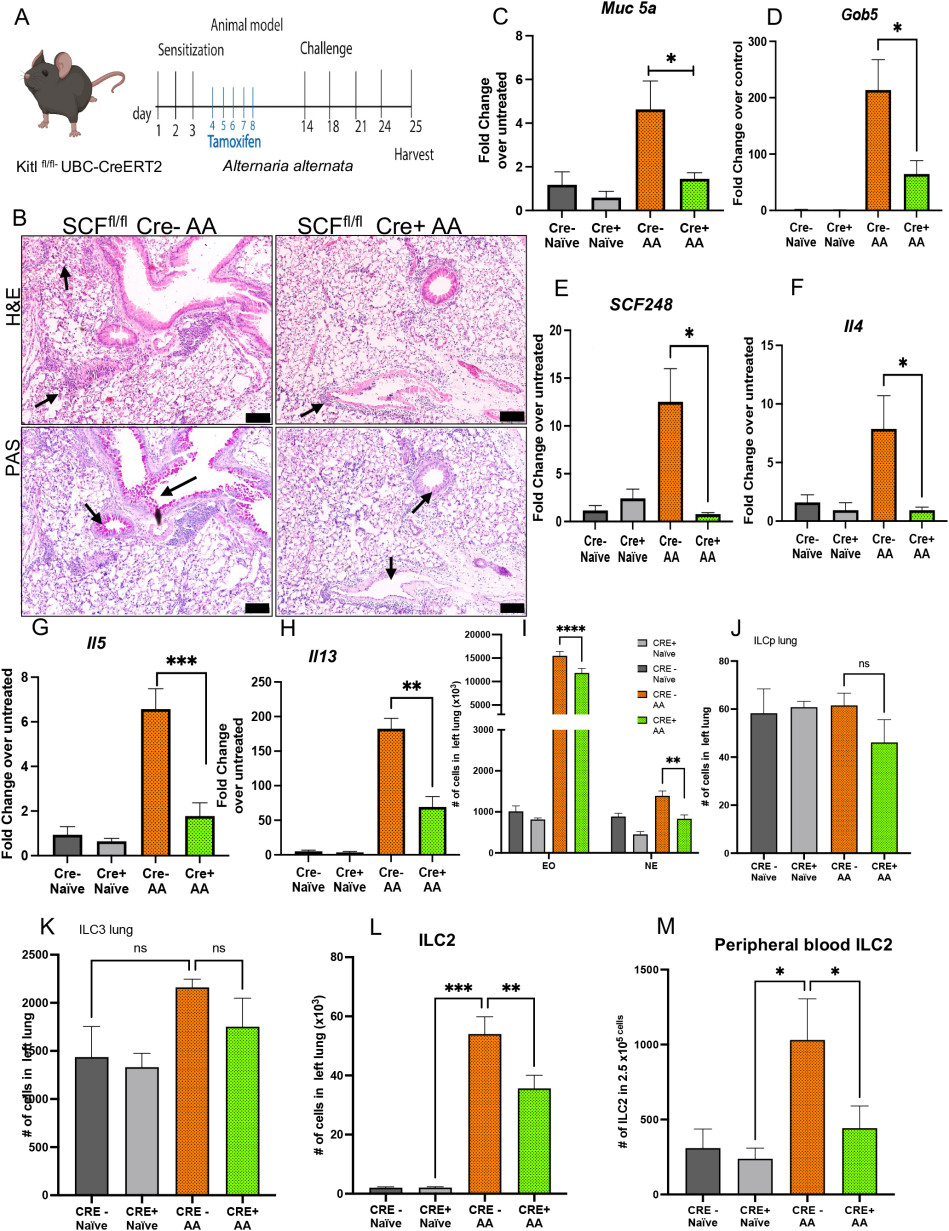
Allergic airway inflammation is attenuated in SCF-deficient mice. **(A)** Animal model inducible SCF KO during the chronic allergic model. **(B)** Lung histopathology showing airway inflammatory infiltration using hematoxylin and eosin (H&E) and mucus deposition, Periodic acid-Schiff (PAS). Scale bar =100μm. **(C, D)** gene expression of mucus-related genes *Muc5a and Gob5*. **(E)** gene expression analysis of SCF248 in the lung. **(F–H)** gene expression analysis of type 2 cytokines in the lung, *Il4*, *Il5* and *Il13*. I-M) Flow cytometry analyses of immune cells, eosinophils (EO), neutrophils (NE), ILCp, ILC3, and ILC2 in the lung, as well as ILC2 detected in samples of peripheral blood. Data are presented as mean ± SEM. Experiments were performed twice, each with 8–10 mice per group (n = 8–10 per group per experiment). Statistical significance was determined using ordinary two-way ANOVA. *p < 0.05; **p < 0.01; ****p < 0.001. NS, not significant.

To evaluate the role of SCF during chronic allergic airway inflammation, SCF^fl/fl^ mice were intranasally sensitized and challenged with *Alternaria alternata.* All mice were treated with tamoxifen intraperitoneally after sensitization, to allow the sensitization process to be established (**Figure 2A**). We used Cre^-^ mice as controls (SCF^fl/fl^Cre^-^). We observed that SCF^fl/fl^Cre^+^ mice had significantly lower responses to allergen challenges, including decreased airway leukocyte infiltration and mucus deposition, compared to SCF^fl/fl^Cre^-^ (**Figure 2B**). These data were corroborated by gene expression of mucus-related genes, with similar patterns observed (**Figures 2C, D**). We have previously observed that the SCF248 proinflammatory isoform is upregulated during allergic responses (58). Here, SCF^fl/fl^Cre^-^ mice highly expressed the SCF248 proinflammatory isoform in the lung, and, as expected, expression was reduced in SCF^fl/fl^Cre^+^ mice (**Figure 2E**). Interestingly, the SCF^fl/fl^Cre^+^ mice exhibited significantly attenuated type 2 lung inflammation compared with SCF^fl/fl^Cre^-^, with decreased expression of *Il4, Il5*, and *Il13* genes in the lung (**Figures 2F–H**). SCF^fl/fl^Cre^+^ showed overall diminished leukocyte numbers in the lung compared to SCF^fl/fl^Cre^-^, with significantly reduced numbers of eosinophils and neutrophils (**Figure 2I**), with no difference in the numbers of pulmonary ILCp and ILC3 (**Figures 2J, K**). However, pulmonary ILC2, as well as ILC2 numbers in peripheral blood samples, were markedly reduced (**Figures 2L, K**). These data demonstrate a critical role of SCF expression during chronic pulmonary allergic disease in the establishment of a type 2 immune response and in the activation and recruitment of ILC2 into the lung, which together contribute to exacerbated allergic disease.

### Upregulation of the SCF248 proinflammatory isoform during chronic allergic asthma in bone marrow impacts hematopoietic ILCp and ILC2 numbers

**Figure 3:**
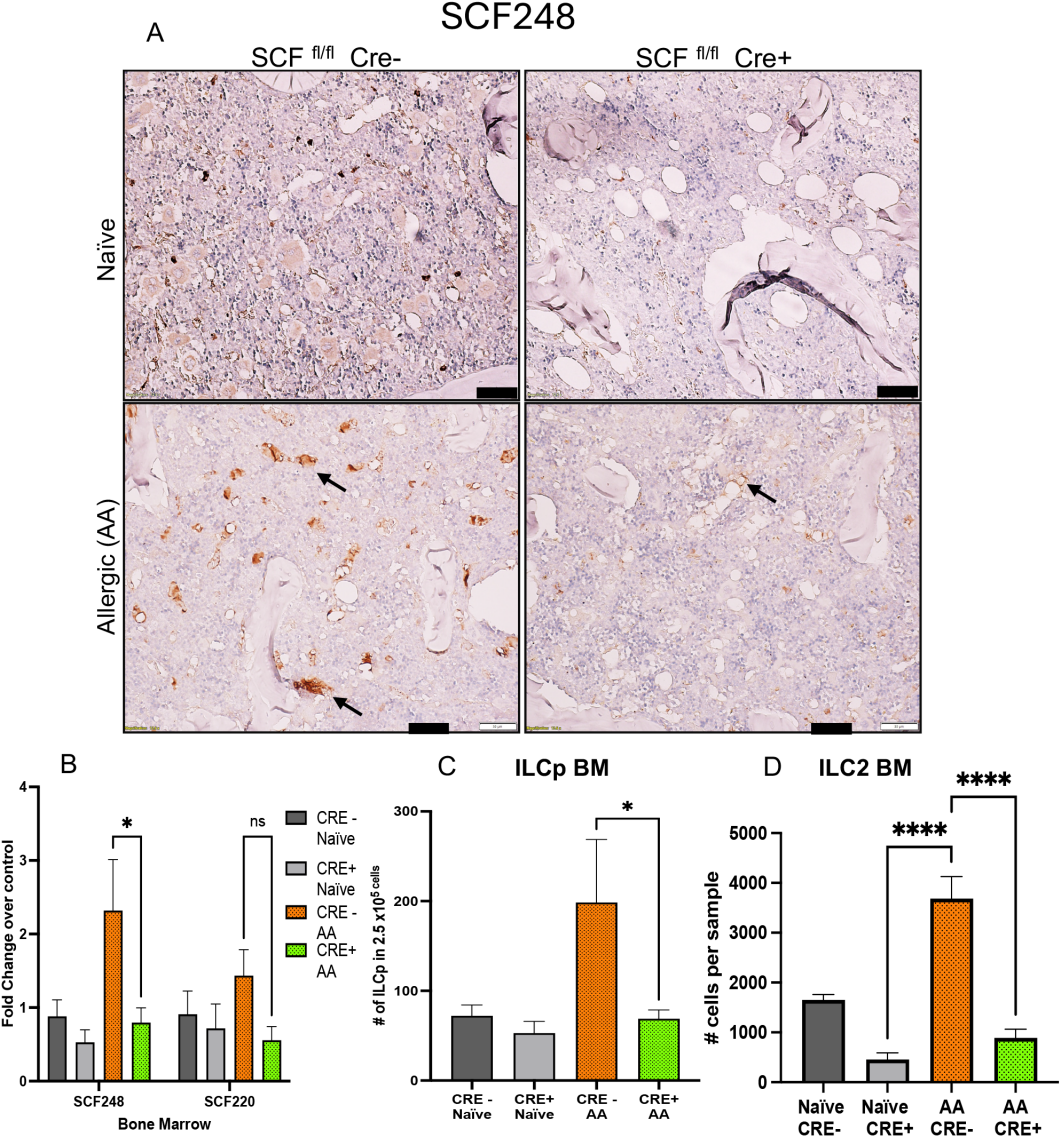
Upregulation of SCF248 proinflammatory isoform during chronic allergy in bone marrow impacts ILCp to ILC2 development. **(A)** Bone marrow immunohistochemistry detecting SCF48 level (dark brown positive staining). Scale bar =100μm. **(B)** Bone marrow gene expression analysis by qPCR detecting SCF isoforms, *SCF220*, and *SCF248*. **(C)** Identification of ILCp in bone marrow by flow cytometry. Data are presented as mean ± SEM. Experiments were performed twice, each with 8–10 mice per group (n = 8–10 per group per experiment). Statistical significance was determined using ordinary two-way ANOVA. *p < 0.05; ****p < 0.001. NS, not significant.

Here, we investigated SCF248 expression in the bone marrow of naïve and chronic allergic mice by immunohistochemistry. SCF248 was detected at low levels in the bone marrow of SCF^fl/fl^Cre^-^ naïve mice and was highly upregulated in allergic mice, with positive immunostaining observed (**Figure 3A**). We verified these data by harvesting bone marrow from naïve and allergic mice and measuring SCF248 expression. We observed that SCF^fl/fl^Cre^-^ allergic mice showed significantly upregulated SCF248 expression but not the SCF220 isoform, compared with naïve and SCF^fl/fl^Cre^+^ mice (**Figure 3B**). Next, ILCp numbers were evaluated in the bone marrow of these mice, and we observed that SCF^fl/fl^Cre^-^ allergic mice, which highly upregulated SCF248, had significantly increased numbers of ILCp cells compared with SCF^fl/fl^Cre^+^ allergic mice (**Figure 3C**). These data highlight the critical role of SCF, specifically the SCF248 isoform, in ILCp-to-ILC2 differentiation during chronic pulmonary allergic responses, indicating that SCF may be a critical cytokine for the maturation, activation and maintenance of ILC2.

### Blocking SCF248 isoform attenuated chronic allergic asthma and impacted hematopoietic ILCp to ILC2

**Figure 4:**
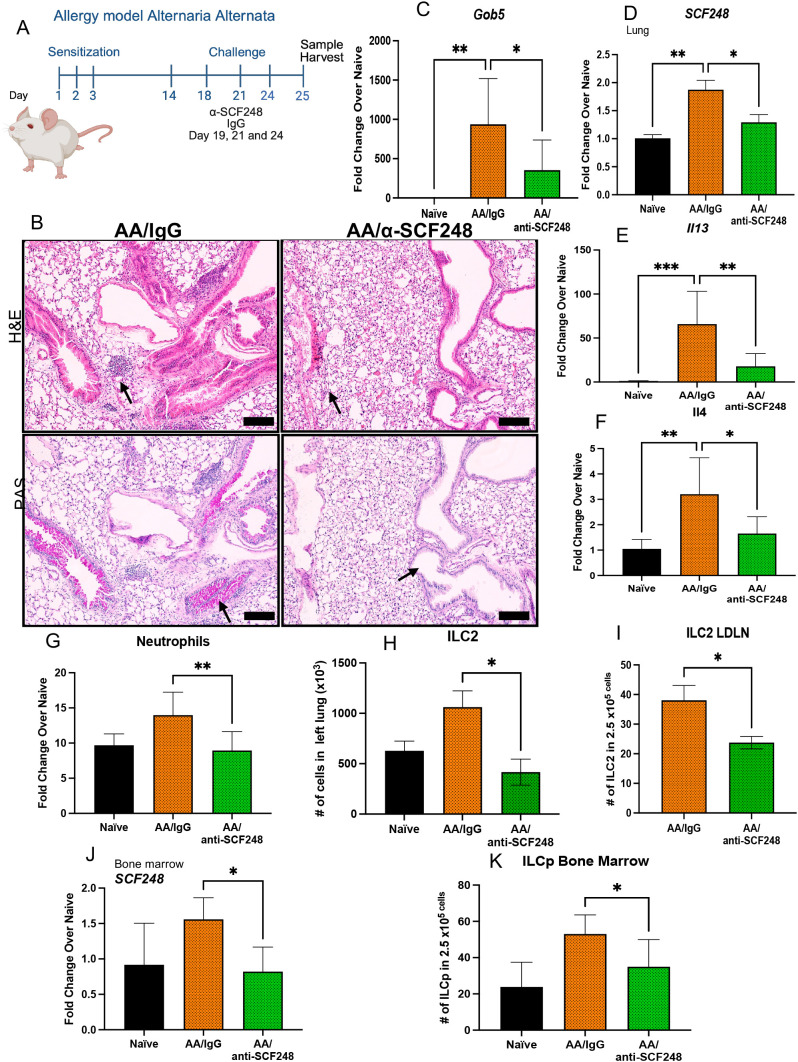
Blocking SCF248 isoform attenuated chronic allergic asthma and impacted hematopoietic ILCp to ILC2 differentiation. **(A)** Animal model of chronic allergy with treatment of anti-SCF248. **(B)** Lung histopathology showing airway inflammatory infiltration using hematoxylin and eosin (H&E) and mucus deposition, Periodic acid-Schiff (PAS). Scale bar =100μm. **(C)** Gene expression of mucus-related gene *Gob5* and D) *SCF248* proinflammatory isoform in the lung. **(E, F)** Gene expression of type 2 cytokines in the lung *Il13* and *Il4*. Flow cytometry analysis of **(G, H)** leukocytes in the lung, neutrophils, and ILC2 cells, as well as I) ILC2 in the lung draining lymph node. **(J)** Bone marrow gene expression analysis of SCF248 proinflammatory isoform and **(K)** ILCp detection by flow cytometry. Data are presented as mean ± SEM. Experiments were performed twice, each with 8–10 mice per group (n = 8–10 per group per experiment). Statistical significance was determined using ordinary one-way ANOVA. *p < 0.05; **p < 0.01; ****p < 0.001.

To understand the overall impact of blocking the SCF248 isoform during chronic asthma, we used the previously described chronic allergic model of *Alternaria alternata* in Balb/c mice and treated the mice with a monoclonal antibody against SCF248 (α-SCF248) or an isotype IgG control antibody (**Figure 4A**). We observed that mice treated with α-SCF248 showed an overall attenuated allergic response, with diminished lung histopathology, significantly reduced expression of mucus-related genes, and reduced expression of the type 2 cytokines *il4* and *il13* in the lung, compared to IgG-control allergic mice (**Figures 4B–F**). Significantly decreased numbers of inflammatory cells, including neutrophils and pulmonary ILC2, were observed in the α-SCF248 group, as well as in lung-draining lymph nodes (**Figures 4G–I**). Interestingly, blocking SCF248 during chronic allergic response alters SCF248 expression in the bone marrow of allergic mice and significantly reduces the number of hematopoietic ILCp. These results suggest that SCF, specifically the SCF248 isoform, plays an important role in hematopoietic ILCp-ILC2 maturation and maintenance of ILC2 activation during chronic pulmonary allergy.

### SCF248 promotes ILC2 maturation and functional programming

**Figure 5:**
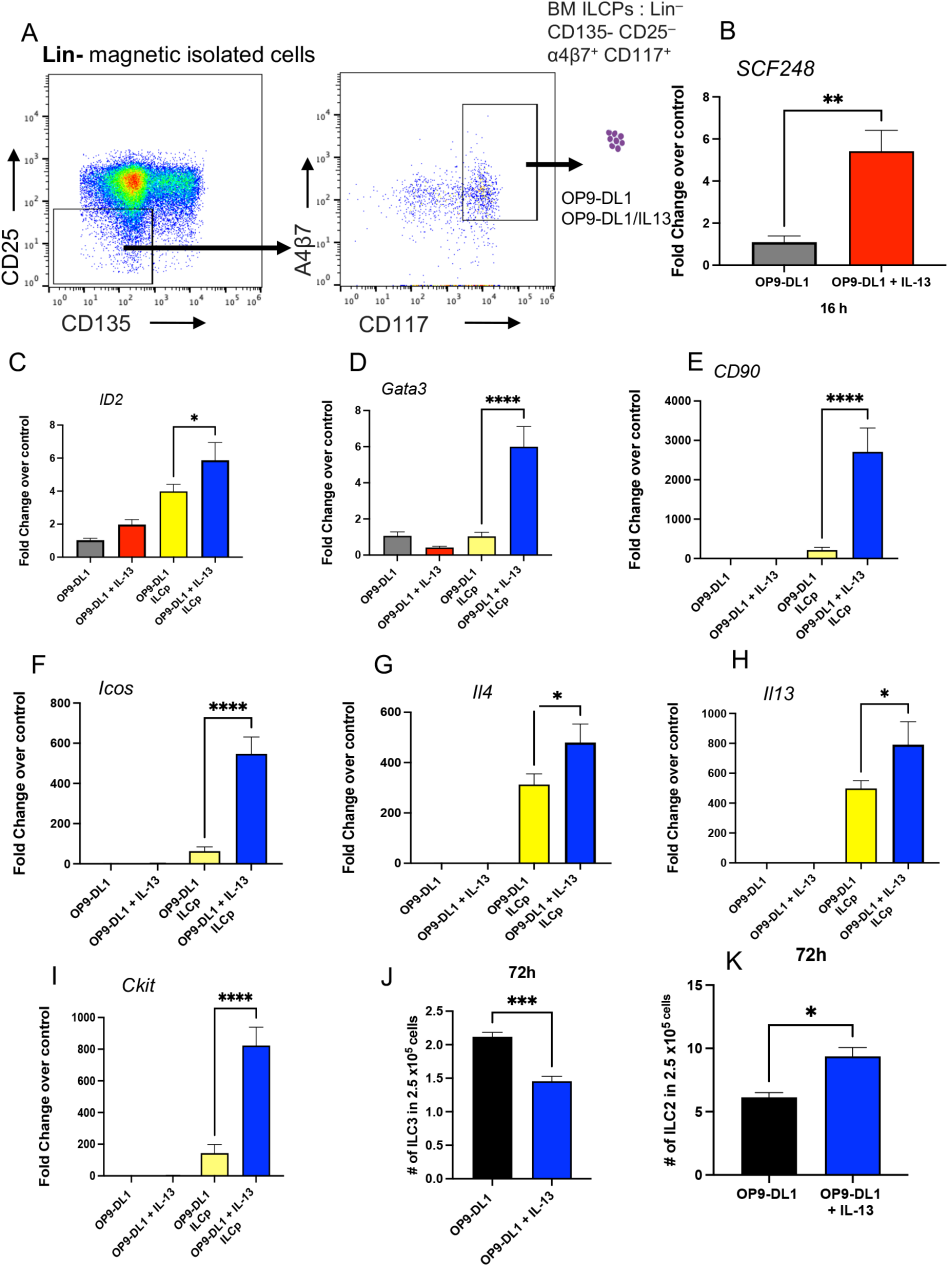
SCF248 expression impacts ILCp-ILC2 lineage specification *in vitro*. Bone marrow ILCp were sorted from naive Balb/c male mice. **(A)** ILCp sorting strategy. **(B)** SCF248 proinflammatory isoform was upregulated in OP9DL1 cells treated with rmIL-13 (10ng/mL for 16h). Gene expression analysis by qPCR was performed on ILCp cells differentiated *in vitro* for 72 h on OP9-DL1 cells or OP9-DL1 cells treated with IL-13. Cells were washed prior to co-culture. **(C-I)** Inhibitor of DNA binding 2 (*ID2*), *Gata3*, *CD90*, *Icos*, *c-Kit*, *Il4*, *Il13*. **(J)** flow cytometry analysis of differentiated ILC2. Data are presented as mean ± SEM. Experiments were performed twice with n = 3–4 replicates per group. Statistical significance was determined using ordinary two-way ANOVA. *p < 0.05; **p < 0.01; ***p < 0.001; ****p < 0.001.

To dissect the role of SCF in the ILC2 lineage specification and identity maintenance. We sorted ILCp cells (**Figure 5A**) and differentiated them *in vitro* using the OP9 delta1 (OP9DL1) cell system (59). The ILCp gene expression phenotype was assessed prior to initiating *in vitro* cultures. Sorted ILCp displayed reduced expression of *C-Kit* and *Gata3* (**Supplementary Figures 1A, B**), as well as undetectable levels of *ICOS*, *CD90*, and *Bcl11b*, markers, and a key transcription factor required for mature ILC2 differentiation. In contrast, ILCp showed increased expression of *Ets1* compared with mature ILC2 cells (**Supplementary Figures 1C–F**). Together, these data confirmed that the ILCp population was accurately identified and sorted. Previously, we observed that pro-inflammatory and type 2 cytokines induced SCF248 expression in mesenchymal cells, supporting its association with allergic airway inflammation. IL-13 produced the strongest induction, providing the rationale for its use in our *in vitro* model (58, 60). Moreover, OP9DL1 upregulated the isoform SCF248 upon exposure to rmIL-13 (**Figure 4B**), rmIL-1β,and rmIL-33 (10ng/mL) (**Supplementary Figures 2A, B**), suggesting that SCF248 expression in mesenchymal cells is induced by proinflammatory and type-2 cytokines. To study ILCp differentiation and activation, we have established an *in vitro* system following published protocols (59, 61). ILCp: Lin-CD25- C-kit+ CD135- α4β7+ (62), were sorted from a single cell suspension of bone marrow, harvested from naïve adult mice. The sorted cells were seeded over OP9-DL1 or OP9-DL1+IL13 (OP9DL1-SCF248). We observed that ILCp cultured on OP9-DL1+IL-13, SCF248 expressing cells, showed significantly increased expression of the transcription factors *ID2* and *Gata3* (**Figures 5C, D**), as well as ILC2 markers such as *CD90* (Thy-1), *ICOS, and c-KIT* (**Figures 5E, G**), and type 2 cytokines, specifically *Il4* and *Il13*, compared to control ILCp cultured on unstimulated OP9-DL1 cells (**Figures 5H, I**). Finally, ILC2 numbers were evaluated by flow cytometry, and we observed a significant increase in ILC2 numbers in the OP9DL1+IL13/ILCp culture system (**Figure 5J**) and a decrease in ILC3 numbers. To directly address the effect of SCF248 upregulation in ILCp, we performed SCF248 blockade by adding α-SCF248 antibody to the cell culture. We observed that neutralization of SCF248 during ILCp differentiation prevented the upregulation of ILC2-associated markers and inhibited the accelerated generation of mature and activated ILC2 cells. (**Supplementary Figures 3A, 4E**). These findings suggest that elevated SCF248 expression in OP9-DL1+IL-13 cells promotes ILCp differentiation toward an ILC2 phenotype and enhances ILC2 maturation, effector programming, and functional competence. 

## Discussion

**Figure 6:**
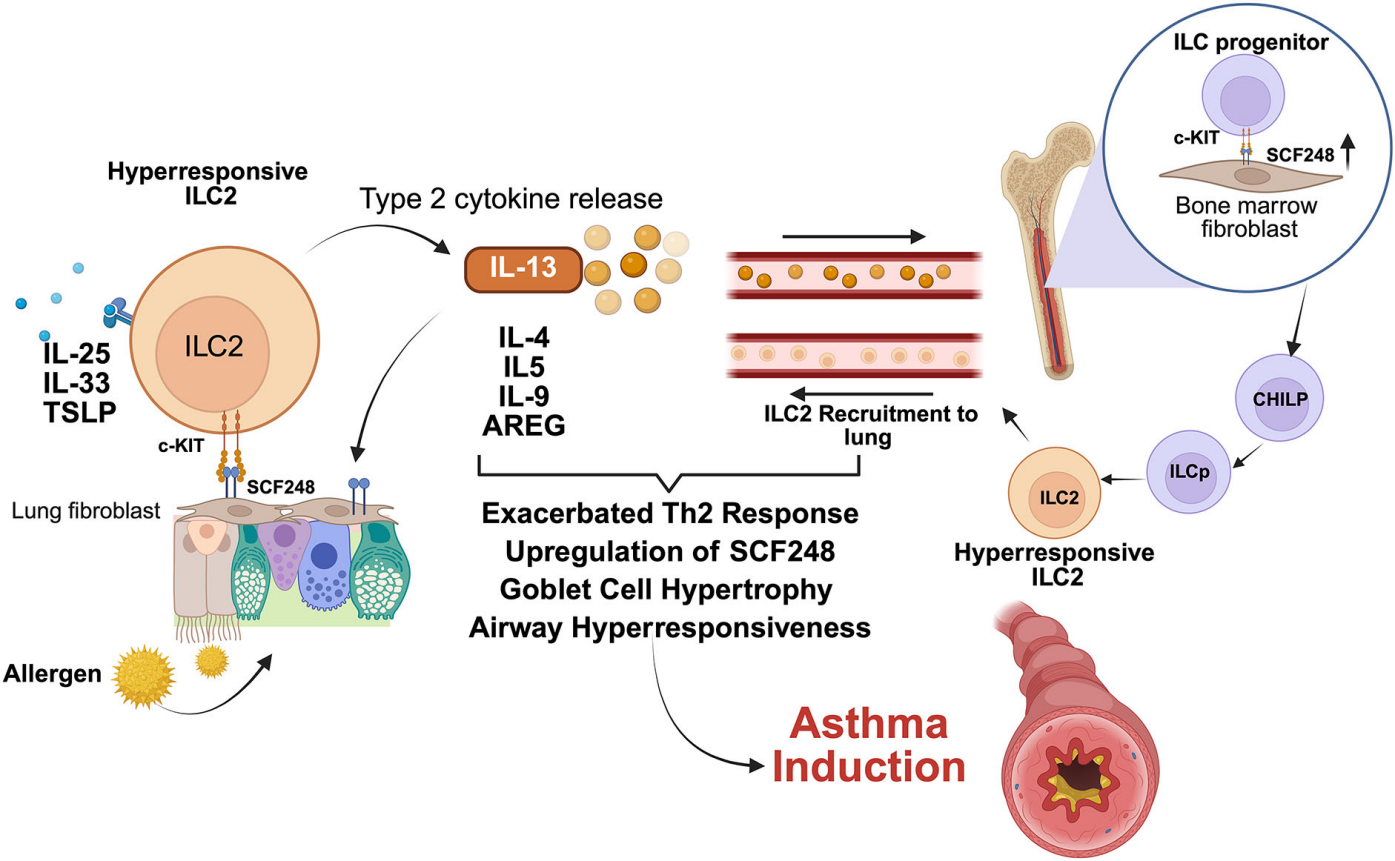
SCF drives ILC2 differentiation during allergy. During allergic type-2 inflammation, SCF expression increases with a shift toward the pro-inflammatory SCF248 isoform in the bone marrow and lung. SCF248 signaling through c-Kit supports the expansion of hematopoietic ILC progenitors and promotes the accumulation of circulating ILC2 that migrate to the lung. Within the lung mucosa, epithelial- and mesenchymal-derived SCF248 enhances tissue ILC2 activation and type-2 cytokine production, including IL-5 and IL-13, driving eosinophilia and mucus metaplasia. These cytokines further induce SCF248 expression in stromal cells, establishing a feed-forward loop that amplifies type-2 inflammation and promotes hyperresponsive ILC2 during allergic airway disease. Created in BioRender.

These studies demonstrate that the SCF248 isoform is upregulated during type 2 inflammation in both the bone marrow and the lung. Acting through c-Kit on ILCp, SCF248 enhances ILC2 maturation in the bone marrow, expanding the pool of circulating ILC2s that can be recruited to the lung during chronic allergic inflammation. Locally, increased SCF248 expression in the lung further activates tissue-resident ILC2s to produce IL-5 and IL-13, driving eosinophilia and mucus metaplasia. These type 2 cytokines, in turn, amplify SCF expression in both the lung and bone marrow, establishing a self-reinforcing feed-forward loop that sustains type 2 inflammation (**Figure 6**).

Transcriptional analysis of ILC2s was conducted in an acute airway inflammatory model designed to capture early activation programs during the initiation of type 2 inflammation. In this model, we did not detect a reduction in the number of hematopoietic ILC2s in the absence of SCF. However, we identified multiple transcriptional changes that collectively indicate impaired effector competency, proliferation, and cytokine responsiveness, accompanied by metabolic remodeling. In naïve SCF-deficient mice, several genes associated with early activation readiness were downregulated, including the immediate-early transcription factors *Fos* and *Fosb* (31, 32). We also observed reduced expression of genes associated with ILC2 activation and signaling competence, including *Icos*, a well-established marker of mature ILC2s that promotes survival and cytokine production (36), as well as components of cytokine signaling pathways such as *Jak1* and *Pik3cg* (37) (38). Additionally, *Arg1*, a metabolic regulator that supports ILC2 proliferation and type 2 inflammatory responses, was decreased (40). During allergic inflammation, SCF-deficient ILC2 displayed reduced expression of key effector and signaling genes, including *Il4* and *Stat5b*, suggesting diminished type 2 cytokine output and impaired responsiveness to cytokine signals required for ILC2 expansion and function (43, 44, 48, 49). Concomitant reductions in signaling intermediates such as *Sh2b1*, *Akt2*, and *Prkcd* further suggest attenuation of PI3K-AKT-associated activation pathways that normally support ILC2 survival and effector programming (52, 54, 56). In contrast, increased expression of *Acadvl*, a regulator of mitochondrial long-chain fatty acid β-oxidation, suggests altered metabolic programming with an enhanced reliance on lipid oxidation (57).

*In vitro* differentiation of hematopoietic ILC progenitors cultured over OP9-DL1-SCF248 cells induced a distinct ILC2-associated transcriptional program characterized by increased expression of c-Kit, ICOS, CD90, GATA3, Id2, and the type 2 cytokines Il4 and Il13. These transcriptional changes were accompanied by significantly higher numbers of mature ILC2s compared with control OP9-DL1 cultures. Together, these findings from both scRNA-seq and *in vitro* differentiation studies support a model in which SCF248 promotes ILC2 maturation, expansion, activation, and effector fitness by enhancing their inflammatory and cytokine-producing capacity, while not being strictly required for ILC2 lineage differentiation.

Increased numbers of ILC2s have been reported in asthmatic patients (3, 63). ILC2s produce substantial amounts of IL-5 and IL-13 in response to epithelial cell-derived cytokines such as IL-25, IL-33, and thymic stromal lymphopoietin (TSLP), which are key drivers in the pathogenesis of allergic diseases, including asthma (64). Notably, challenge with *Alternaria alternata* induces high IL-33 expression in the airway epithelium, leading to activation of ILC2s and recruitment of ILC progenitors from the bone marrow to the lung (65–67). In our model, SCF deletion or blockade of SCF248 isoform markedly reduced ILC2 activation and recruitment, even in the presence of high IL-33 levels in the lung. Although IL-25, IL-33, and TSLP are key upstream activators of ILC2s, SCF-c-Kit signaling provides complementary signals for survival, expansion, and metabolic support. In SCF-deficient conditions, reduced cellular fitness and proliferative capacity likely limit full responsiveness to these cytokines, preventing complete compensation.

Furthermore, SCF220 is constitutively expressed and remains relatively stable, including during inflammation, whereas SCF248 is strongly and selectively upregulated in the airway and bone marrow during allergic inflammation. Both isoforms signal through c-Kit, but their distinct expression patterns suggest different functional roles in ILC biology (20–22). In our model, induction of the proinflammatory isoform SCF248 correlates with enhanced ILC2 activation and maturation, while SCF220 likely maintains baseline c-Kit signaling.

Serum SCF levels are elevated in patients with asthma and correlate with disease severity (68, 69). Soluble SCF is generated by proteolytic cleavage of the SCF248 isoform, which contains a cleavage site absent in the homeostatic SCF220 isoform. This structural distinction suggests that SCF248 is preferentially upregulated during asthmatic allergic inflammation in humans.

SCF is well known for its role in hematopoiesis and mast cell maturation, survival, activation, and degranulation. SCF also enhances IgE-dependent responses and promotes airway hyperreactivity and inflammation in experimental asthma models (12, 17, 70). Previous studies have shown that SCF is upregulated during chronic allergic inflammation in the lung and that blocking the SCF248 isoform reduces the numbers of c-Kit^+^ cells, including eosinophils, mast cells, and ILC2s (58). A limitation of the SCF-deficient animal model used in this work is that we cannot fully exclude the contribution of other c-Kit+ cells, such as mast cells, *in vivo.*

Human circulating ILC2s exhibit heterogeneous c-Kit expression, which has been associated with distinct functional states: c-Kit^low^ ILC2s are generally considered more mature and effector-like, whereas c-Kit^high^ ILC2s may display greater responsiveness and potential plasticity under specific inflammatory conditions. Importantly, SCF signaling has been shown to enhance type 2 cytokine production in ILC2s (71), suggesting that SCF-c-Kit interactions may support ILC2 activation and functional programming. These findings align with our data, which demonstrate that SCF248 promotes ILC2 maturation and activation rather than lineage flexibility. Nonetheless, the precise cause-and-effect relationship between SCF signaling and human ILC2 function in asthma remains to be fully elucidated.

SCF is unlikely to be the sole factor regulating ILC2 differentiation, maturation and activation and we recognize that multiple cytokines and environmental cues contribute to this process, however, in this work we observed that SCF248 upregulation during allergy accelerated maturation of ILC2 and hyperactive the cells, suggesting an important role of SCF248 during ILC2 related pulmonary disease.

In conclusion, here we observed that the proinflammatory isoform SCF248 is highly upregulated during chronic allergic inflammation, not only in the lung but also in the bone marrow. SCF deficiency or blockade of the SCF248 isoform in a mouse model of allergic asthma resulted in attenuated pulmonary type 2 immune responses, reduced accumulation of inflammatory ILC2s in the lungs, and improved overall disease outcomes. Importantly, SCF-deficient mice showed reduced numbers of ILC progenitors in the bone marrow, as well as decreased ILC2 in circulation and in the lung, demonstrating a role for SCF in ILC2 maturation and activation. Together, our findings show that SCF, particularly SCF248, promotes ILC2 maturation and phenotypic tuning. By enhancing hematopoietic ILC2 maturation, effector programming, and mucosal activation states, SCF248 amplifies type 2 immune responses and may represent a potential therapeutic target for limiting ILC2-driven pulmonary inflammation.

## Materials and methods

### Animals

The University of Michigan Institutional Animal Care & Use Committee approved all experiments involving the use of animals. Male Balb/c mice (Strain #000651; RRID: IMSR_JAX:000651), 6–8 weeks of age, were purchased from The Jackson Laboratory (Bar Harbor, ME). Mice with *loxP* sites on either side of exon 1 of the *Kitl* gene (kit ligand; also called SCF, stem cell factor) (STOCK *Kitl^tm2.1Sjm^*/J) were purchased from Jackson Laboratory (Strain #:017861; RRID: IMSR_JAX:017861). The STOCK *Kitl^tm2.1Sjm^*/J mice were crossed with a mouse carrying a tamoxifen-inducible Cre recombinase (Cre-ERT2) under the control of the human ubiquitin C (UBC) promoter B6.Cg-*Ndor1^Tg(UBC-cre/ERT2)1Ejb^*/1J (Strain #007001; RRID: IMSR_JAX:007001) (72), to obtain the whole-body inducible SCF knockout mice SCF^fl/fl^**^;^**^UBC-cre-ERT^ (SCF^fl/fl^) for this project. Mice from this founder line have strong tamoxifen-inducible *Cre* activity in all tissue types. Standard pathogen-free conditions were maintained in the Unit for Laboratory Animal Medicine at the University of Michigan. Treatment conditions were replicated across various mice cohorts.

### Allergy model

In this work, we used the *Alternaria alternata* allergy model, which is a predominantly IL-33-driven and ILC2-dominant model of type 2 airway inflammation.

For scRNA-seq, we used an acute exposure setting to identify transcriptional changes in the bone marrow ILC2 population. Transgenic mice SCF^fl/fl^Cre^+^ and Cre^-^ mice were treated with tamoxifen peritoneally for 5 days, at day 8, mice were challenged daily (30μg in 30μl of saline) for 5 days with *Alternaria alternata* intranasally, and bone marrow was harvested at day 13.

For the chronic allergic model, the mice were sensitized by intranasal administration of *Alternaria alternata* (AA) (30 µg in 30 µl of saline) under light sedation with isoflurane for three consecutive days, followed by intranasal challenge on days 14, 18, 21, and 24. Samples were harvested after the last challenge. Transgenic mice SCF^fl/fl^Cre^-^ and SCF^fl/fl^Cre^+^ were treated with tamoxifen intraperitoneally for five days (days 4-8, after sensitization). SCF is also essential for erythropoiesis; therefore, the timing of tamoxifen administration was chosen to avoid disruption of steady-state red blood cell turnover and other effects in hematopoietic stem/progenitor cells. Balb/c mice were treated with anti-SCF248 previously characterized (58) or with isotype-matched monoclonal antibody by intraperitoneal injection at a concentration of 20mg/kg on days 19, 21, and 24.

### Single-cell RNA sequencing and data analysis

Bone marrow from allergic and control Kitlfl^/fl-UBC-cre-ERT^ mice was collected by flushing the femur and tibia of the hind legs with RPMI 1640 supplemented with 10% fetal calf serum (FCS), L-glutamine, penicillin/streptomycin, non-essential amino acids, and sodium pyruvate. We combined bone marrow samples from 3 mice per group and sequenced them to assess sample heterogeneity. To enrich the stem precursor cells and mesenchymal population in the bone marrow samples, we magnetically isolated mature l cells, including T cells, B cells, monocytes/macrophages, granulocytes, erythrocytes, and their committed precursors, using a Direct Lineage Cell Depletion Kit, and following the manufacturer’s instructions (Miltenyi Biotec). Subsequently, one-third of the isolated Lineage+ cells were reintroduced into the original sample. scRNA-seq was performed following previously published protocols (30, 73). scRNA-seq was performed on ~10,000 cells per sample using the Chromium Next GEM Single Cell 3′ v3.1 platform (10x Genomics) at the University of Michigan Advanced Genomics Core. Raw sequencing data were processed with Cell Ranger using the mm10 mouse reference genome to generate gene–barcode matrices. Downstream analyses were conducted in R (v4.1.0) using Seurat. Cells with 200–5,000 detected genes and <20% mitochondrial transcripts were retained. Data were log-normalized and scaled prior to dimensionality reduction and clustering using a graph-based approach. Cluster-specific marker genes were identified using the Wilcoxon rank-sum test (FindMarkers/FindAllMarkers), and cell-type annotation was performed based on established literature and PanglaoDB. SD and HFD datasets were integrated using Seurat’s anchor-based integration workflow (FindIntegrationAnchors and IntegrateData). Differential gene expression analyses were performed using Seurat and DESeq2, with Benjamini–Hochberg correction for multiple testing. Significant genes were defined as adjusted p < 0.05 and |log2 fold change| > 0.5. Differential expression results are presented as enhanced volcano plots, and selected gene expression patterns are shown as heat maps.

### Quantitative RT-PCR

Lung tissue was homogenized in TRIzol reagent (Thermo Fisher Scientific, Carlsbad, CA), and RNA was extracted according to the manufacturer’s recommendations (Invitrogen, Carlsbad, CA). cDNA was synthesized using murine leukemia virus reverse transcriptase (Invitrogen, Carlsbad, CA) and incubated at 37 °C for one hour, followed by incubation at 95 °C for 10 min to stop the reaction. Real-time quantitative PCR (qPCR) was multiplexed using TaqMan primers with a FAM-conjugated probe all from Thermo Fisher Scientific (Sup. **Table 1**). Fold change was quantified using the 2^−ΔΔ^cycle threshold (CT) method. Custom primers were designed to measure *Muc5ac* and *Gob5* mRNA levels as described before (74). All reactions were run on a 7500 Real-Time PCR System (Applied Biosystems, Foster City, CA).

### Lung histology

The left lung was fixed with 4% (vol/vol) formaldehyde and embedded in paraffin. Five-micrometer lung sections were stained with periodic acid-Schiff (PAS) to detect mucus production and inflammatory infiltrates. Photomicrographs were captured using a Zeiss Axio Imager Z1 and AxioVision 4.8 software (Zeiss, Munich, Germany).

### Flow cytometry

The lungs, lung-draining lymph nodes, and bone marrow were harvested on day 25 to analyze the different leukocyte populations by flow cytometry, following our published protocol (58, 75). In brief, lung and LDLN single cells were isolated by enzymatic digestion (58, 75). Bone marrow was collected by flushing the femur and tibia of the hind legs with RPMI 1640 supplemented with 10% fetal calf serum (FCS), L-glutamine, penicillin/streptomycin, non-essential amino acids, sodium pyruvate, and filtered. Cells were resuspended in PBS, and live cells were identified using a LIVE/DEAD Fixable Yellow Dead Cell Stain kit (Thermo Fisher Scientific, Waltham, MA), then washed and resuspended in PBS with 1% FCS and Fc receptors were blocked with purified anti-CD16/32 (clone 93; BioLegend, San Diego, CA). Surface markers were identified using Abs (clones) against the following antigens, all from BioLegend: anti-Gr-1 (RB6- 8C5), B220 (RA3-6B2), CD3 (145-2C11), Ter119 (Ter-119), CD11b (M1/70), CD25 (PC61), CD45 (30-F11), c-Kit (2B8), CD90 (53-2.1), NKp46 (29A1.4), CD135 (A2F10) and α4β7 (DATK32). For innate lymphoid cell type 2 (ILC2) staining, lineage markers were anti-CD3, CD11b, B220, Gr-1, and TER119. ILC2: Lin-CD45+ CD90+ICOS+c-Kit+ ILCPs: Lin^–^ CD135- CD25^–^ α4β7^+^ CD117^+^. ILC3: Lin-CD45+CD90+c-Kit+NKp46+. Data was collected using a NovoCyte flow cytometer (ACEA Bioscience, Inc., San Diego, California). Data analysis was performed using FlowJo software (Tree Star, Oregon, U.S.A.).

### Immunohistochemistry for SCF in mouse femur sections

Immunohistochemical staining for SCF was performed on 4-µm–thick serial sections of decalcified, paraffin-embedded femur samples. Femurs were fixed in 10% neutral buffered formalin (Sigma-Aldrich, St. Louis, MO) overnight at 4 °C, followed by decalcification in 14% EDTA, pH 7.4–7.6 (Sigma-Aldrich) at room temperature for 2 weeks. Samples were processed and embedded in paraffin using standard protocols. Paraffin sections were deparaffinized in xylene (Fisher Scientific, Waltham, MA) and rehydrated through graded ethanol solutions to phosphate-buffered saline (PBS, Gibco, Thermo Fisher Scientific). Endogenous peroxidase activity was blocked using 3% hydrogen peroxide (Sigma-Aldrich) for 10 min. Non-specific binding was blocked with 1% bovine serum albumin diluted in PBS for 1 h at room temperature. Sections were incubated overnight at 4 °C with the primary monoclonal antibody anti-SCF248 (58). After washing, sections were incubated with an HRP-conjugated goat anti-mouse IgG secondary antibody (Vector Laboratories, Burlingame, CA) for 30–60 min at room temperature. Antigen visualization was performed using a DAB peroxidase substrate kit (ImmPACT DAB, Vector Laboratories), producing a brown reaction product detectable under bright-field microscopy. Sections were counterstained with Mayer’s hematoxylin (Sigma-Aldrich), dehydrated, cleared in xylene, and mounted with Permount mounting medium (Fisher Scientific). Negative controls were processed in parallel by omitting the primary antibody.

### ILC progenitors isolation and differentiation

Bone marrow from naive male Balb/c mice was collected by flushing the femur and tibia of the hind legs with RPMI 1640 supplemented with 10% fetal calf serum (FCS), L-glutamine, penicillin/streptomycin, non-essential amino acids, and sodium pyruvate. We combined bone marrow samples of 10–15 mice. Next, we enriched our progenitor population by magnetically isolating Lineage^+^ cells using a Direct Lineage Cell Depletion Kit and following the manufacturer’s instructions (Miltenyi Biotec). The enriched Lineage negative cells were stained against CD135 ^(PE)^, CD25 ^(Pacific Blue)^, α4β7 ^(APC)^, CD117 ^(PeCy7)^, and FACS sorted (BD FACS Discover S8 sorter), selecting CD135- CD25^–^ α4β7^+^ CD117^+^. The sorted cells were seeded in 96-well plates over OP9-DL1 or OP9-DL1+IL13 stromal cells (OP9DL1-SCF248), which had been previously treated with rmIL-13 (10ng/ml) and washed to remove IL-13 from the culture after 16h of stimulation. We used OP9-DL1 and OP9-DL1+IL13 (OP9DL1-SCF248) cells alone as controls to establish baseline expression of the analyzed genes. The ILCp cells were cultured in RPMI supplemented with 10% FBS, glutamine, penicillin, streptomycin, and 30ng/mL of recombinant murine IL-7 for 72 hours at 37 C and 5% CO_2._ To block SCF248 in OP9DL1+IL13 SCF248-expressing cells, we added anti-SCF248 antibody (10 µg/mL) to the culture 20 min before adding ILCp cells. RNA was extracted to measure gene expression, and flow cytometry was performed to identify mature ILC2.

### Statistical analysis

Data were analyzed using GraphPad Prism 10 (GraphPad Software). Results are presented as mean ± SEM. Comparisons between two groups were performed using unpaired, two-tailed Student’s t-test. Comparisons among three groups were analyzed using one-way ANOVA with Tukey’s *post hoc* test. For experiments involving two independent variables (four-group designs), two-way ANOVA was used followed by appropriate multiple comparisons testing. A p-value < 0.05 was considered statistically significant.

## Data Availability

The data presented in the study are deposited in the NCBI GEO repository, accession number GSE336792.
