# Supplementary material for: Stem cell factor 248 shapes ILC2 transcriptional programs and promotes mucosal inflammation in allergic asthma
Source: Front Immunol. 2026 Jul 2;17:1843080. doi: 10.3389/fimmu.2026.1843080 (PMC13372909; doi:10.3389/fimmu.2026.1843080)
Supplement: Supplementary file 1 [file Table1.docx]

***Supplementary Figure S1.***

******

***Supplementary Figure S1.* Bone marrow ILCp phenotype.** Gene expression analysis of sorted ILCp cells compared with mature *in vitro* differentiated ILC2. A-F) *c-Kit, Gata3, Icos, CD90, Bc11b,* and *Etsl*. Data are presented as mean ± SEM. n = 4 replicates per group. Statistical significance was determined using an unpaired t-test. *p < 0.05; ****p** < 0.01; ******p** < 0.001.

***Supplementary Figure S2.***

***
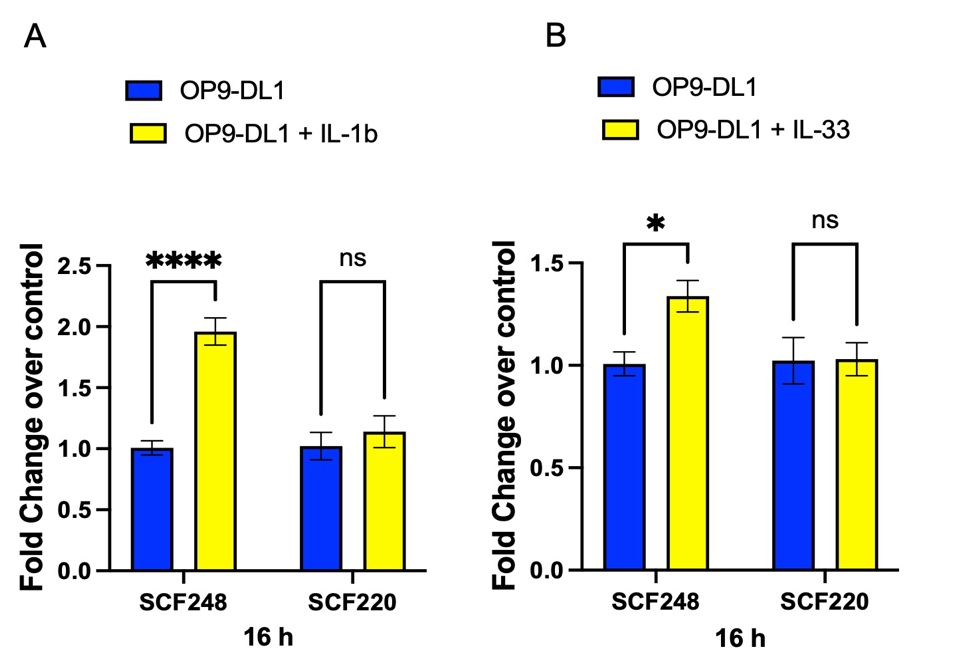
***

***Supplementary Figure S2.* SCF isoforms gene expression in OP9-DL1 cells.** Gene expression analysis of OP9-DL1 cells treated with rm cytokines A) IL-1b and B) IL-33. Data are presented as mean ± SEM. n = 4 replicates per group. Statistical significance was determined using ordinary two-way ANOVA. *p < 0.05; ******p** < 0.001.

***Supplementary Figure S3.***

***
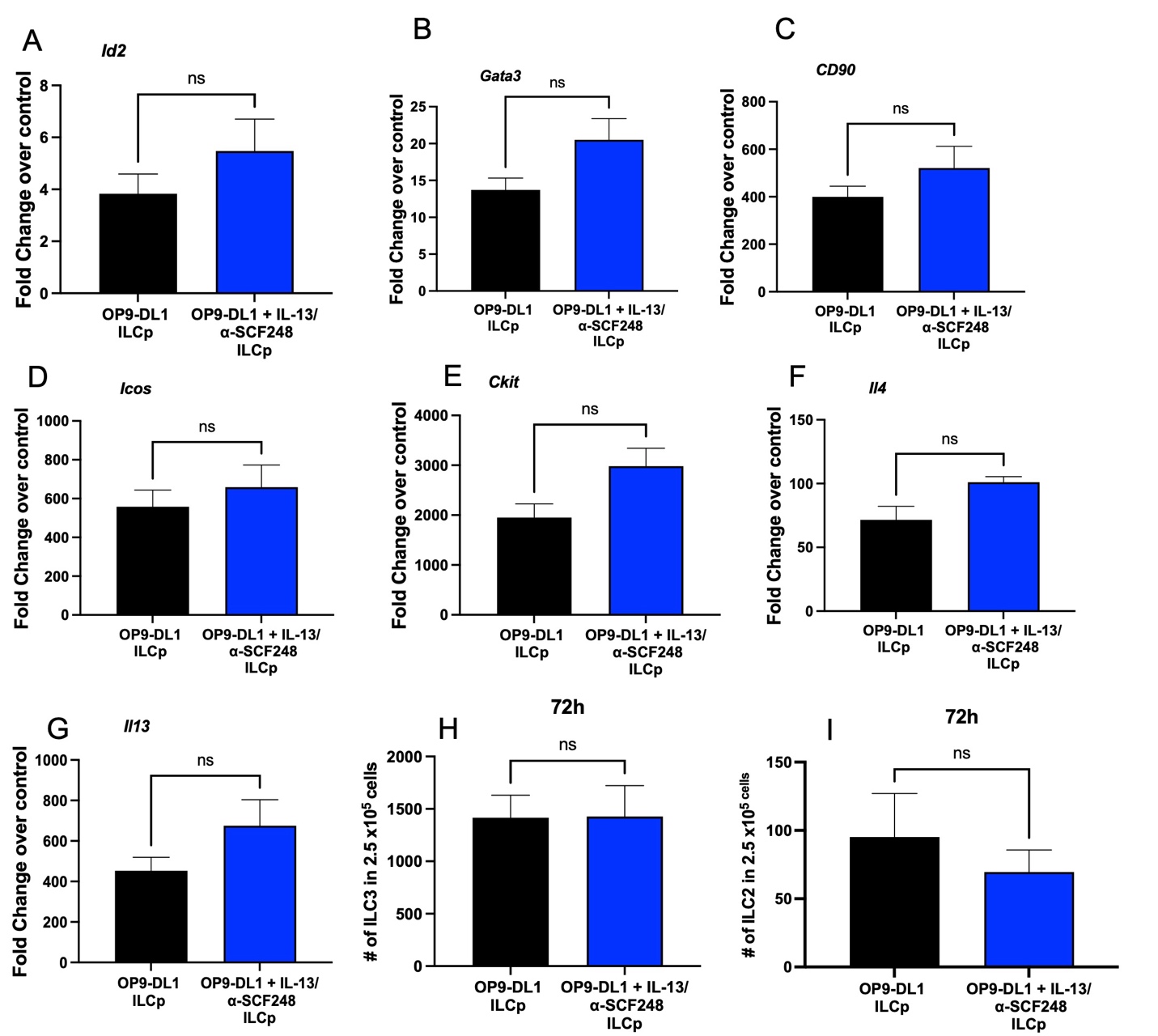
***

***Supplementary Figure S3. Blocking SCF248 reduces the magnitude of ILC2 activation and expansion in vitro.*** Bone marrow ILCp were sorted from naive Balb/c male mice and differentiated in vitro. Gene expression analysis by qPCR was performed on ILCp cells differentiated in vitro for 72 h on OP9-DL1 cells or OP9-DL1 cells treated with IL-13. Cells were washed prior to co-culture. A- G) Inhibitor of DNA binding 2 (*ID2*), *Gata3*, *CD90*, *Icos*, *c-Kit*, *Il4*, *Il13*. H-I) flow cytometry analysis of differentiated ILC2 and ILC3. Data are presented as mean ± SEM. Experiments were performed twice with n = 3-4 replicates per group. Statistical significance was determined using unpaired t-test.

***Sup.Table 1. Primers for gene expression analysis***

| ***Gene*** | **Assay ID Taq Man** |
| --- | --- |
| *Rn18s* | Mm03928990_g1 |
| *il4* | Mm00445260_m1 |
| *il5* | Mm00439646_m1 |
| *il13* | Mm00434204_m1 |
| *id2* | Mm00711781_m1 |
| *Gata3* | Mm00484683_m1 |
| *CD90(thy1)* | Mm00493681_m1 |
| *Icos* | Mm00497600_m1 |
| *c-Kit(kit)* | Mm00445212_m1 |
